# Effective NiMn Nanoparticles-Functionalized Carbon Felt as an Effective Anode for Direct Urea Fuel Cells

**DOI:** 10.3390/nano8050338

**Published:** 2018-05-16

**Authors:** Nasser A. M. Barakat, Mohannad Alajami, Zafar Khan Ghouri, Saeed Al-Meer

**Affiliations:** 1 Organic Materials and Fiber Engineering Department, College of Engineering, Chonbuk National University, Jeonju 561-756, Korea; Mhndalajame@hotmail.com; 2 Chemical Engineering Department, Faculty of Engineering, Minia University, Minia 61111, Egypt; 3 Central Laboratories Unit, Qatar University, P. O. Box 2713, Doha, Qatar; zafarkhanghouri@hotmail.com (Z.K.G.); salmeer@qu.edu.qa (S.A.-M.)

**Keywords:** functionalized gas diffusion layer, direct urea fuel cell, urea electrooxidation

## Abstract

The internal resistances of fuel cells strongly affect the generated power. Basically, in the fuel cell, the anode can be prepared by deposition of a film from the functional electrocatalyst on a proper gas diffusion layer. Accordingly, an interfacial resistance for the electron transport is created between the two layers. Electrocatalyst-functionalized gas diffusion layer (GDL) can distinctly reduce the interfacial resistance between the catalyst layer and the GDL. In this study, NiMn nanoparticles-decorated carbon felt is introduced as functionalized GDL to be exploited as a ready-made anode in a direct urea fuel cell. The proposed treated GDL was prepared by calcination of nickel acetate/manganese acetate-loaded carbon felt under an argon atmosphere at 850 °C. The physiochemical characterizations confirmed complete reduction for the utilized precursors and deposition of pristine NiMn nanoparticles on the carbon felt fiber. In passive direct urea fuel cells, investigation the performance of the functionalized GDLs indicated that the composition of the metal nanoparticles has to be optimized as the GDL obtained from 40 wt % manganese acetate reveals the maximum generated power density; 36 mW/m^2^ at room temperature and 0.5 M urea solution. Moreover, the electrochemical measurements proved that low urea solution concentration is preferred as utilizing 0.5 M solution resulted into generating higher power compared to 1.0 and 2.0 M solution. Overall, this study opens a new avenue toward functionalization of the GDL as a novel strategy to overcome the interfacial resistance between the electrocatalyst and the GDL.

## 1. Introduction

Urea-contaminated water represents a big environmental challenge due to the inevitable treatment. Environmentally, emission of the formed ammonia gas from urea hydrolysis is a main dilemma required an urgent treatment [1]:

NH_2_CONH_2_ + H_2_O → 2NH_3_ + CO_2_ (1)

Moreover, there are two groups of bacteria (*Nitrobacter and Nitrosomonas*) having the ability to oxidize the water-soluble ammonia to nitrate (NO_3_) with an unstable intermediate nitrogen oxide (NO_2_) product [2]. This process goes on under anoxic conditions where the nitrate ion is reduced to several nitrous gases. In addition to the aforementioned problems, urea pollution can trigger ocean algae to produce a deadly toxin called domoic acid [3]. Considering the large amounts of polluted water discharged from urea fertilizer manufacturing plants (0.75 m^3^ wastewater containing around 1 wt % urea per ton of fertilizer produced) [4], and animal and human urines, the treatment process becomes very costly.

Fortunately, urea can be manipulated as a hydrogen storage material due to the fact it embeds a considerable amount of hydrogen (6.67 wt %). Two methodologies were proposed to exploit the hydrogen content present in urea and simultaneously eliminate this environmentally undesirable compound; electrolysis and electro-oxidation in a direct urea fuel cell. In electrolysis, hydrogen can be obtained through electrochemical oxidation according to the following reactions [5,6,7]:

Anode:

CO(NH_2_)_2_ + 6OH^−^ → N_2_ + 5H_2_O + CO_2_ + 6e^−^, E^0^ = −0.746 V (2)

Cathode:

6H_2_O + 6e^−^ → 3H_2_ + 6OH^−^, E^0^ = −0.829 V (3)

Overall:

CO(NH_2_)_2_ + H_2_O → N_2_ + 3H_2_ + CO_2_, E^0^ = −0.083 V (4)

The electrolysis process is not economically preferable due to the very high anode overpotential this distinctly decreases the overall cell potential and consequently increases the required electrical energy to perform the oxidation process even with utilizing the precious metals [8].

Theoretically, urea can be exploited as an effective fuel in a direct urea fuel cell (DUFC) with relatively high cell potential compared to some direct alcohol fuel cells according to the following reactions [9]:

Anode:

CO(NH_2_)_2_ + 6OH^−^ → N_2_ + 5H_2_O + CO_2_ + 6e^−^, E^0^ = −0.746 V (5)

Cathode:

3H_2_O + 1.5O_2_ + 6e^−^ → 6OH^−^, E^0^ = +0.40 V (6)

Overall:

CO(NH_2_)_2_ + 1.5O_2_ → N_2_ + 2H_2_O + CO_2_, E^0^ = +1.146 V (7)

However, the very high onset potential for most of the reported electrocatalysts constrains practical application in DUFCs. In this regard, several reported materials including pristine metals, oxides and hydroxides showed very good electrocatalytic activity toward urea oxidation; however, these materials cannot be exploited to prepare anode for the DUFCs. In other words, the observed anode potentials are very high compared to the standard urea oxidation potential that makes the final cell potential negative, which means it is not thermodynamically feasible. Therefore, developing a suitable anode material to shift the process to be thermodynamically passable (i.e., positive cell potential) can lead to establish a direct urea fuel cell to generate power during electro-oxidation of the urea.

Precious metals (e.g., Pt) are widely used as excellent performance anode materials for direct alcohol fuel cells, however these metals show poor activity toward urea electro-oxidation [10]. Nickel-based materials also offer a good chance to be exploited as anode materials in urea electrolysis and sometimes in the direct urea fuel cells [10,11,12]. However, the corresponding high onset potential (ca. 0.45 V vs. SHE) compared to the theoretical one exemplifies a big constraint toward a real application in the fuel cell. Several trials have been carried out to develop effective electrodes based on morphology modification including nickel nanoparticles [13], nickel nanowires [11], nickel nanoribbons [14], and nickel-carbon sponge [15] or exploiting the synergetic effect of the Ni-based alloys such as Ni-Mn [12], Ni-Co [5], and Ni-Zn [4]. However, utilizing the co-catalyst reveals better improvement compared to the morphology modification [8,12,16,17,18].

Besides the high anode overpotential, the cell internal resistances strongly decrease the generated power. There are several internal resistances in the fuel cell including mainly the membrane resistance, the interfacial resistance between the anode and cathode functional material and the gas diffusion layers (GDLs), and the interfacial resistance between the GDL and current collectors [19]. Typically, to prepare the anode (or the cathode), an ink composed from the function electrocatalyst, filler (e.g., carbon black) and binder (e.g., Nafion) is used to form a thin layer over the GDL (e.g., carbon cloth, carbon paper or carbon felt). Accordingly, an interfacial resistance for the electron transfer is created between the electrocatalyst layer and the GDL or the current collector. Therefore, if the electrocatalyst could be chemically deposited on the surface of the GDL to prepare a readymade anode without using a binder and filler, this can distinctly decreases the interfacial resistance that would result in enhancing the generated power. Moreover, synthesis the electrocatalyst in the form of nanoparticles bonded with the GDL can strongly facilitate the electron transfer process.

In this study, a novel NiMn-nanoparticles-functionalized GDL is introduced to overcome the interfacial resistance at the anode and consequently enhances the generated power. Briefly, the carbon felt which have very good characteristics as GDL (excellent porosity, high electrical conductivity, distinguished chemical stability, and very acceptable mechanical properties) was decorated by NiMn nanoparticles and utilized as a ready made anode in a direct urea fuel cell. The decoration process was achieved by deposition of the metal acetates of the chosen metals and then calcination under an inert atmosphere at high temperature. The electrochemical measurements of the assembled fuel cells indicated that the nanoparticles composition and urea solution concentration should be optimized to maximize the obtained power; 40 wt % Mn content and 0.5 M urea solution exhibit the highest power density.

## 2. Experimental Work

### 2.1. Preparation of the Functionalized GDL

Due to the known hydrophobicity of the carbon felt (Alfa Aesar, Seoul, Korea), the utilized metals precursors (nickel (II) acetate tetrahydrate, NiAc, 98%, Aldrich Co., St. Louis, MO, USA, and manganese (II) acetate tetrahydrate, MnAc, 99%, Aldrich Co.) were dissolved in absolute ethanol (SamChun Chemicals Co., Seoul, Korea) rather than the distilled water. Typically, 0.1 g total metals acetates was well dissolved in 3 mL ethanol, stirring was carried out until clear solution was obtained. Then, using a syringe, the prepared solution was gently poured on 3 cm × 3 cm carbon felt sheet after careful washing by acetone to remove any impurities in the carbon felt. The solution was poured in steps; each time 1 mL was poured and then the sheets were subjected to drying process at 80 °C for 30 min which was enough for a complete evaporation of the ethanol. After complete pouring of the prepared solution and evaporation of the used solvent, the treated carbon felt sheets were calcined under argon atmosphere at 850 °C for 3 h. Therefore, due to absence of oxygen, the carbon felt was not affected by the calcination process; however, the abnormal decomposition of the acetate under the oxygen-free atmosphere led to form pristine metals. To investigate the metallic nanoparticles composition, different samples were prepared by changing the MnAc content with respect to NiAc; 0 wt %, 5 wt %, 10 wt %, 20 wt %, 40 wt %, 60 wt %, 80 wt % and 100 wt % MnAc were used.

### 2.2. Direct Urea Fuel Cell Structure

Passive fuel cell with a 23 mL fuel tank was used to check the performance of the introduced functionalized carbon felt GDL sheets. Based on the utilized characterizations, which confirm complete reduction of the utilized precursors, the catalyst loading was estimated as 2.67 mg/cm^2^. The cathode was 2.5 cm × 2.5 cm Pt-loaded (0.5 mg/cm^2^) carbon paper (EC-20-5, Electro Chem, Inc., Woburn, MA, USA). Anion exchange membrane (AEM, Membrane International Inc., Ringwood, NJ, USA) was used. The membrane was treated by dipping in an KOH solution (0.5 M) for 18 h, and then kept in a distilled water. The current collectors were two gold-plated 0.1 cm thick high corrosion resistance stainless steel plates. Different urea (Alfa Aesar) solution concentrations were used; 0.5, 1.0 and 2.0 M in 1 M KOH. Measurement of the open circuit potentials and the generated power was controlled by VersaState poteniostate (AMETEK, Berwyn, PA, USA). The current density and power density were normalized to the net anode area exposed to the solution; 4.4 cm^2^.

### 2.3. Characterizations

Surface morphology was studied by scanning electron microscope (SEM, JSM-5900, JEOL, Peabody, MA, USA) and field-emission scanning electron microscope equipped with energy dispersive X-ray (EDX) analysis tool (FESEM, S-7400, Hitachi, Tokyo, Japan). Information about the phase and crystallinity was obtained by using Rigaku X-ray diffractometer (XRD, Rigaku, Tokyo, Japan) with Cu Kα (λ = 1.540 Å) radiation over Bragg angle ranging from 10° to 100°. The electrochemical measurements were achieved by VersaStat 4 poteniostate (Princeton Applied Research-AMETEK, Oak Ridge, TN, USA) using the VersaStudio software (Princeton Applied Research-AMETEK, Oak Ridge, TN, USA).

## 3. Results and Discussion

Ethanol has good wettability for carbonaceous materials; moreover, it has a good solubilization power for several ionic salts. Accordingly, the utilized metals acetates could be dissolved in the ethanol; a clear solution was obtained which led to well distribution of the used precursors over the carbon sheets. Based on our previous studies and others, it was proved that calcination of the transition metals acetates under inert atmosphere results in complete reduction of the salt to produce pristine metals rather than obtaining the oxides forms. For instance, formation of the pure nickel rather than nickel oxides can be described by the following reactions [20,21,22,23]:

Ni(CH_3_COO)_2_·4H_2_O → 0.86Ni(CH_3_COO)_2_·0.14Ni(OH)_2_ + 0.28CH_3_COOH + 3.72H_2_O (8)

0.86Ni(CH_3_COO)_2_·0.14Ni(OH)_2_ → NiCO_3_ + NiO + CH_3_COCH_3_ + H_2_O (9)

NiCO_3_ → NiO + CO_2_ (10)

NiO + CO → Ni + CO_2_ (11)

**Figure 1:**
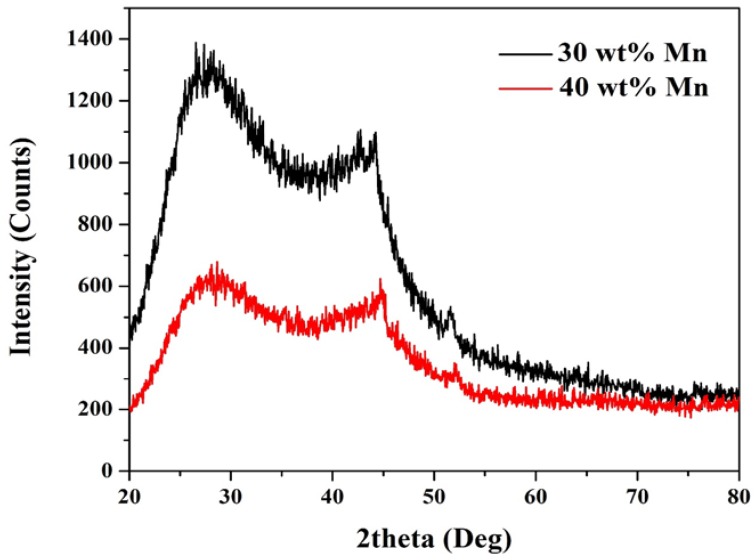
XRD patterns for two treated carbon felt sheets.

**Figure 2:**
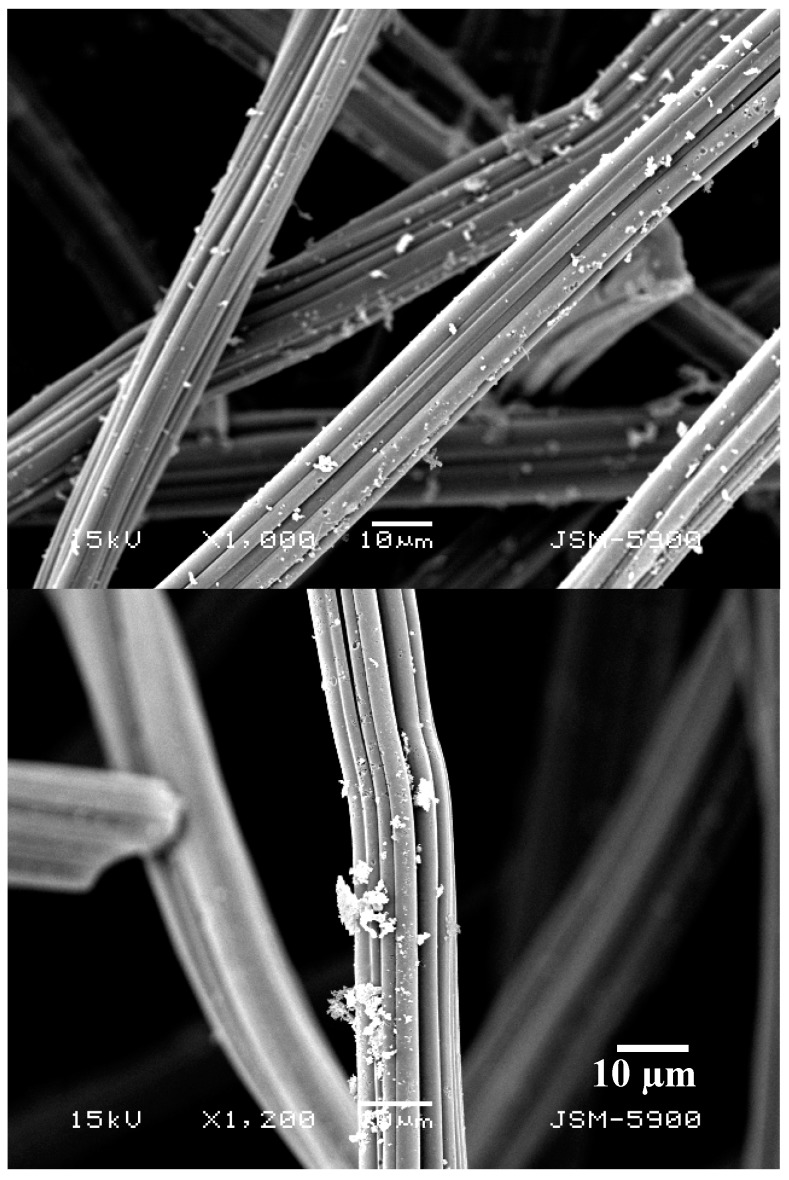
Two magnifications SEM images for the treated carbon felt sheet after the calcination process; sample 40 wt % MnAc.

Although, manganese is more active compared to nickel, the high calcination temperature needed can also result in complete reduction [8]. It is worth mentioning that the complete reduction process for the used acetate precursors to zero-valency metals was achieved due to evolving strong reducing gases (CO and H_2_) from the abnormal decomposition of the acetate ion under the inner gas atmosphere [20]. Accordingly, the appeared CO in Equation (11) was formed from the acetate decomposition [20]. Consequently, the obtained XRD patterns in Figure 1 can be understood. The diffraction peaks appear at 2θ of 44.5°, 51.8° and 76.4° corresponding to (1 1 1), (2 0 0) and (2 2 0) crystal plans, respectively can be assigned to Ni (JCDPS# 04-0850). On the other hand, for the sample obtained from using 30 wt % MnAc, manganese representative peaks can be seen at 2θ of 42.8°, 45.3°, and 76.8° corresponding to (2 2 1), (3 1 0) and (5 1 0) crystal plans, respectively (JCDPS# 33-0887). It is noteworthy mentioning that due to the small amount of the metallic nanoparticles compared to the carbon felt, the intensities of the corresponding peaks are small especially the last one (at 76.4°). Moreover, the discrete distribution of the metallic nanoparticles, as shown in the FESEM image (Figure 2), results in producing wide peaks. Although in the second sample prepared from higher amount of MnAc precursor (40 wt %), the Mn peaks could not be detected. Considering that Mn has high melting point (1246 °C), it is not believed that the formed Mn evaporated. Therefore, it is acceptable to claim that in this sample NiMn alloy has been formed [24]. Additionally, the broad shoulder peak at 2θ of 26.3° corresponding to an experimental *d* spacing of 3.37 Å represents the carbon felt (*d* (0 0 2), JCPDS; 41-1487). No other peaks refer to metal oxides can be detected which indicates full decomposition of the used precursors to the pristine metals, which is supported also by the previous studies. It is noteworthy mentioning that obtaining the broad peaks indicates formation of nano-sized metal nanoparticles.

**Figure 3:**
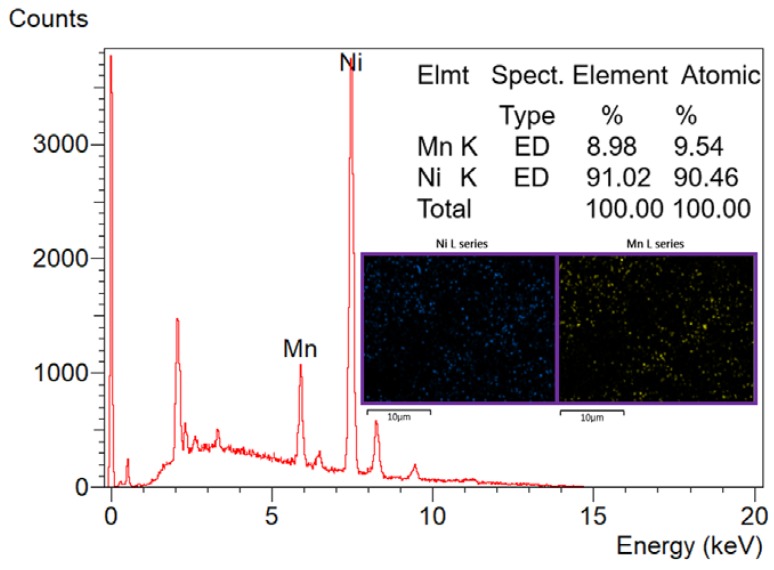
EDX result for the treated carbon felt using 40 wt % MnAc as an initial precursor.

Based on the XRD results, the attached nanoparticles to the carbon felt fibers appeared in the scanning electron microscope images (Figure 2) can be assigned to the metal nanoparticles. The average particle size for the attached nanoparticles was determined to be 775 ± 140 nm. Besides XRD, EDX analysis was performed to check the elemental composition of the treated carbon felt. As shown in Figure 3, Ni and Mn peaks appears clearly which supports the final description of the treated sheets as NiMn nanoparticles-decorated carbon felt. The inset in Figure 3 demonstrates elemental mapping for the attached nanoparticles. As shown, both metal have almost the same distribution, which indicates formation of MnNi alloy and simultaneously supports the XRD results.

Simplicity and wide applicability are the most important advantages of the passive fuel cells compared to the active ones. Moreover, passive fuel cells can be easily used for the portable devices. However, high mass transfer rate is a very important feature for the active fuel cells which distinctly improves the performance. Moreover, in the active fuel cells, the air humidity at the cathode can be also controlled which enhances the cathode performance and consequently the generated power. Specifically in the direct urea fuel cells, a maximum relative humidity (RH) at the cathode is definitely required to achieve good performance because water is a reactant at the cathode in order to form OH ions (Equation (6)) [25]. In this study, passive fuel cell structure was selected to investigate the performance of the introduced functionalized GDL.

Basically, urea oxidation occurs on the surface of the nickel-based electrocatalysts via the electrochemical NiOOH active layer which can be formed in the alkaline medium. Generally, in the activation voltammogram of the nickel-based electrocatalysts, two regions can be seen; the first region is shown at the negative potential side comprising an anodic peak representing the oxidation of nickel according to the reaction [26]:

Ni + 2OH^−^ → Ni(OH)_2_ + 2e^−^ (12)

The peak representing the aforementioned reaction is very small in the first cycle and disappears in the subsequent ones [26,27,28]. On the other hand, the second region is seen at the positive potential region. Comparatively, this peak is stronger than that the corresponding one for the first reaction, and related to the oxidation of Ni(OH)_2_ to NiOOH according to this reaction [28,29,30]:

Ni(OH)_2_ + OH^−^ → NiOOH + H_2_O + e^−^ (13)

**Figure 4:**
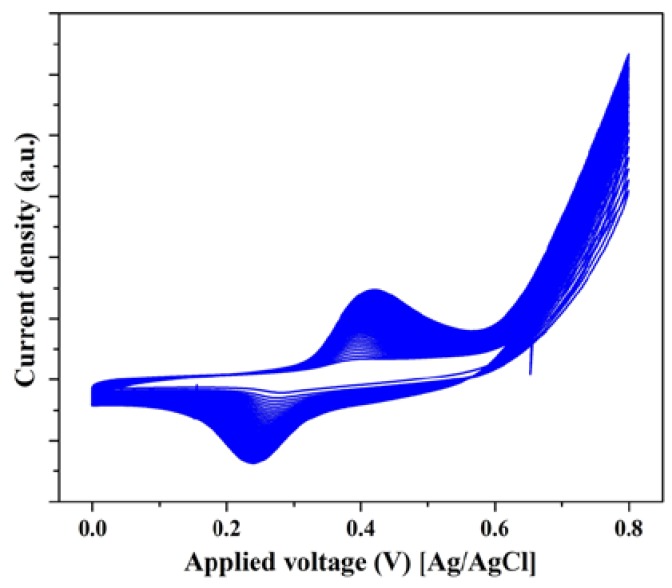
Activation of the NiMn-based electrocatalyst in 1.0 M KOH solution at scan rate of 0.05 V/s.

Increasing the number of potential sweeps leads to a progressive increase of the current density values of the cathodic peak because of the entry of OH^−^ into the Ni(OH)_2_ surface layer, which indicates a progressive formation of the desired NiOOH layer [26]. Therefore, the observed peaks in Figure 4 are attributed to the Ni(OH)_2_/NiOOH transformation reaction.

Accordingly, the alkaline solution concentration can affect the electrocatalyst performance. Vedharathinam and Botte [13] introduced a comprehensive study about the electrooxidation of the urea on the surface of nickel-based materials. It was concluded that the alkaline solution concentration has a strong impact on the current density, especially at the low concentration (lower than 1 M). However, after 1 M KOH concentration, low improvement in the catalyst performance was observed. Accordingly, the concentration of the alkaline medium in this study was kept at 1.0 M.

**Figure 5:**
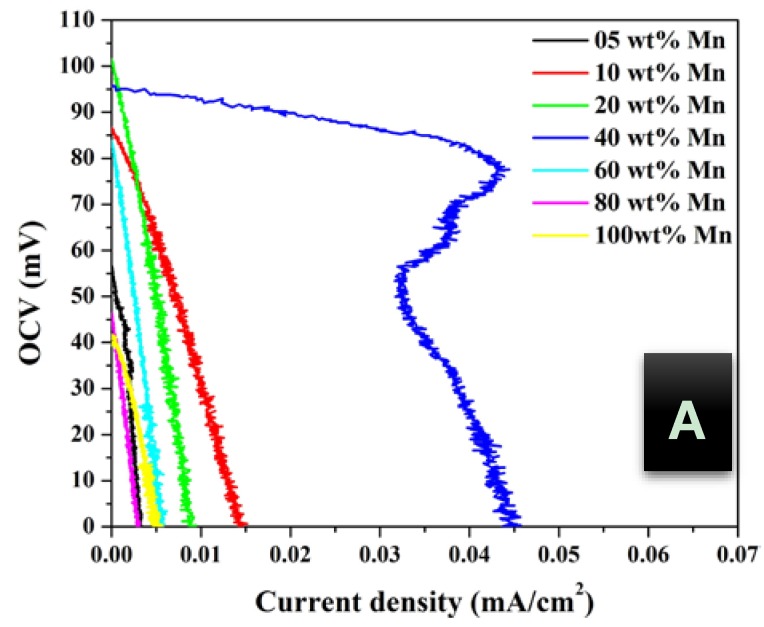

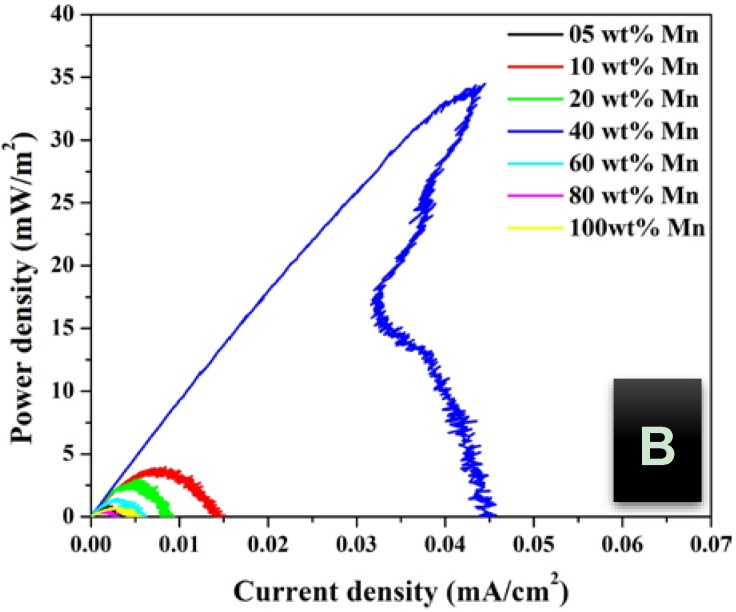
Polarization; (**A**) and power density; (**B**) curves of DUFC at room temperature with 0.5 M urea as fuel using functionalized carbon felt at different metal nanoparticles composition as anode.

**Figure 6:**
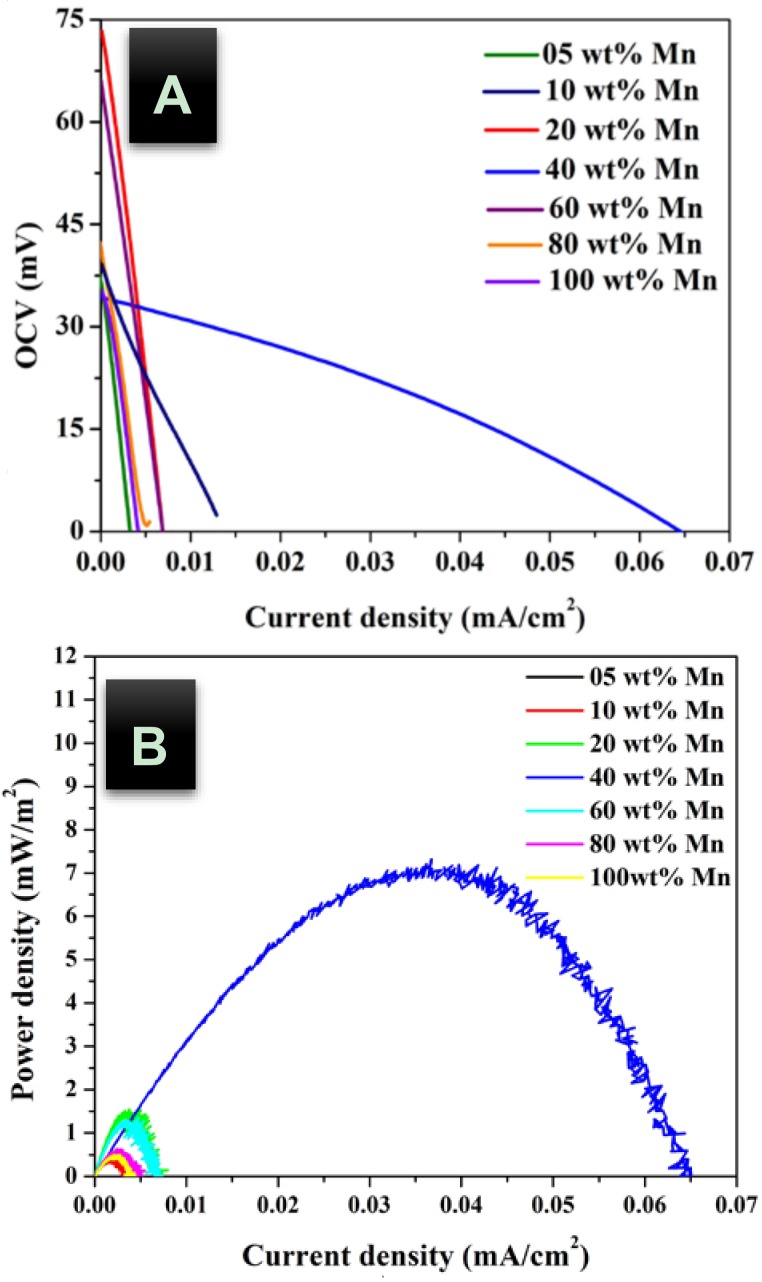
Polarization; (**A**) and power density; (**B**) curves of DUFC at room temperature with 1.0 M urea as fuel using functionalized carbon felt at different metal nanoparticles composition as anode.

**Figure 7:**
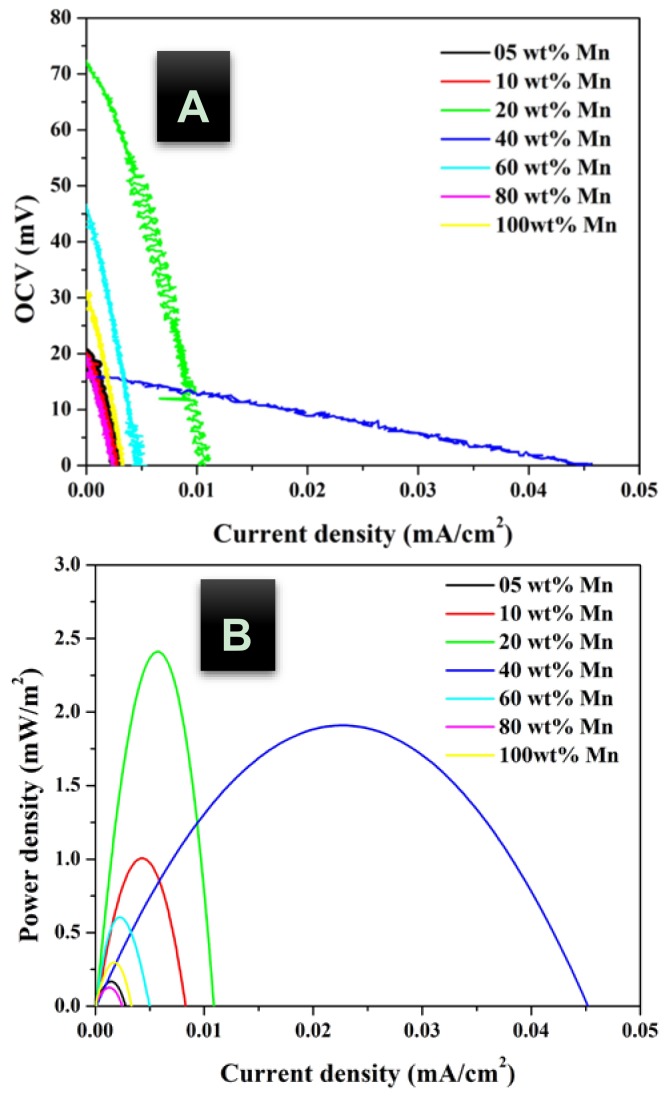
Polarization; (**A**) and power density; (**B**) curves of DUFC at room temperature with 2.0 M urea as fuel using functionalized carbon felt at different metal nanoparticles composition as anode.

In addition, urea concentration should be also investigated to maximize the obtained power. Figure 5, Figure 6 and Figure 7 display the influence of urea concentration on the cell potential and the generated power for different metal nanoparticles compositions. As shown in Figure 5A, a maximum cell potential of 103 mV was obtained with the treated GDL by 20 wt % MnAc. It is known that the commercial anion exchange membrane creates high cell internal resistance that reflects low current densities for all formulations. It is clear that the 40 wt % MnAc sample reveals the maximum current density; 0.045 mA/cm^2^ while the other formulations generate relatively low and close current densities. Figure 5B depicts the generated power from each cell, as shown 36 mW/m^2^ could be produced when the functionalized GDL by 40 wt % MnAc was exploited as anode. Figure 6 and Figure 7 display the created cell potential and the generated power density from all the assembled cells using 1.0 and 2.0 M urea solutions, respectively. As shown, in case of the cell potential, the longest platform was obtained with the 40 wt % MnAc sample which results in the highest maximum current at all urea concentrations. On the other hand, the maximum power density was also obtained with this functionalized GDL except in case of 2.0 M urea solution; Figure 7B. The priority of the 40 wt % sample can be attributed to the detected alloy structure in the XRD analysis. It is known that the alloy composition provides new electronic structure that creates novel characteristics.

Overall, the electrodes’ overpotentials (anode and cathode) and the ohmic resistance are the main parameters behind the fast decrease in the cell voltage upon increasing the current density. Ohmic losses are related to the generated electrical resistances at several parts including the electrolyte solution, the outer circuit and the membrane. Based on the obtained results, for the optimum GDL (40 wt % Mn), compared to the overpotentials, the ohmic resistances do have a high contribution in the cell potential losses. This hypothesis can be proved by the fast decrease in the cell potential with increasing the current [31]. The ohmic resistance is related to the constant voltage drop region at the middle of the polarization curve, which usually consists of three regions [31]. The other two terminal regions appear at relatively low and high current that display rapid voltage losses. Among the aforementioned kinds of resistance, the membrane possesses the maximum value. Therefore, it is believed that kinetic limitation control depends on the activity of the used GDL. Typically, the required overpotential to perform the urea oxidation reaction at the inactive anodes can be assigned as the rate controlling step. On the other hand, the ions transfer resistance through the membrane is the kinetic controlling step when an efficient anode is used.

Table 1 summarizes the estimated cell resistance. As shown, the DUFC assembled by the functionalized GDL corresponding to 40 wt % Mn nanoparticles reveals the lowest resistance at all concentrations. Considering that both of the ohmic resistance and the cathode overpotential are independent on the composition of the bimetallic nanoparticles, the distinct decrease in the resistance for the best GDL-based cell can be assigned to the distinguished decrease in the anode overpotential. Accordingly, these results conclude that the overpotential is the main reason behind the low generated power for the low performance GDLs, and simultaneously support the aforementioned hypotheses about the rate-controlling step. Moreover, it can be also claimed that, the interfacial resistance for the electron transferred, which is considered a part of the anode overpotential, was also decreased using the optimum GDL. Unfortunately, measuring the interfacial resistance is not an easy task so its value is expressed by the overpotential.

**Figure 8:**
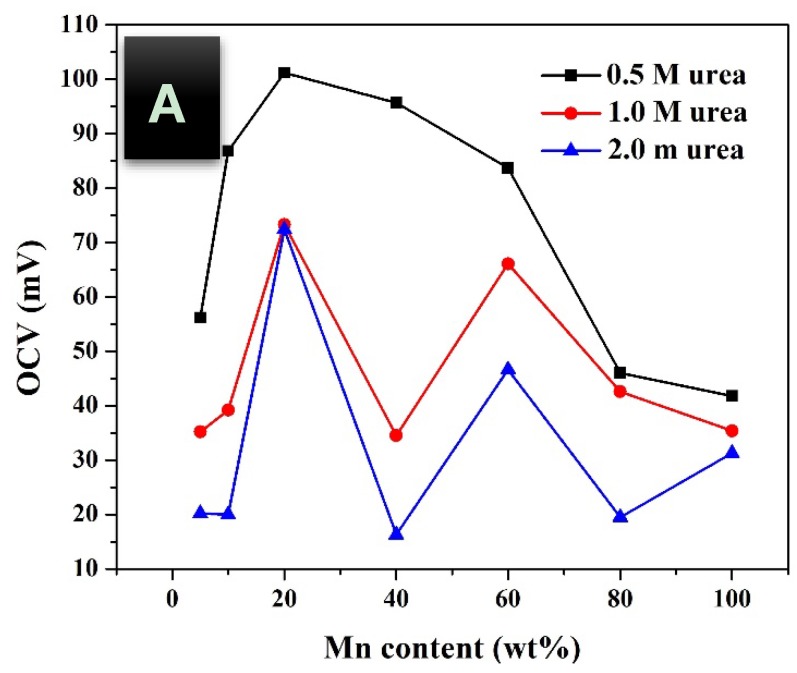

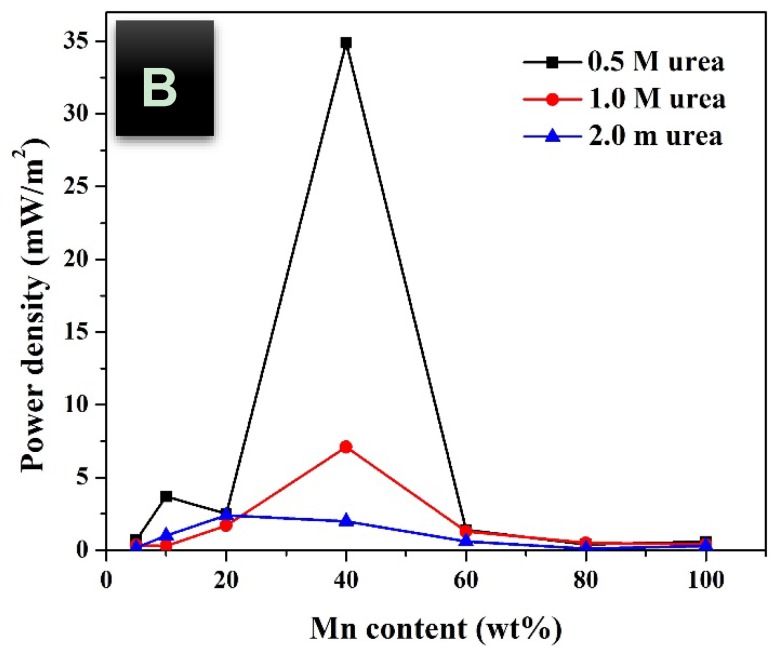
Influence of the metal nanoparticles composition on the obtained cell potential; (**A**) and generated power; (**B**) from direct urea fuel cells using 0.5, 1.0 and 2.0 M urea solutions at room temperature.

The open circuit voltage (OCV) is related to the maximum theoretical voltage can be obtained from the cell before real generation of the power as it is estimated at a zero current. As shown in Figure 8A, with all urea concentration, the maximum OCV are obtained with the functionalized GDL by 20 wt % MnAc precursor that might be leading to conclude that this GDL will generate the maximum current. However, investigation of generated power from the assembled cell reveals a different conclusion. Surprisingly, although the treated carbon felt by 40 wt % MnAc reveals relatively low OCV, the maximum generated power density is attributed to the DUFC containing this functionalized GDL as shown in Figure 8B. Therefore, and based on the obtained results in Table 1, it can be claimed that the overpotential at the anode surface has a distinct impact on the generated power. In other words, although it was expected that 20 wt % sample will lead to generate the maximum power, the corresponding high overpotential changes the real finding. This conclusion was assigned to the anode overpotential because the other parameters affecting the voltage loss are independent on the bimetallic nanopartciles composition as aforementioned. It is noteworthy mentioning that the functionalized GDL by pristine nickel nanoparticles did not give positive cell potential which can be explained by the known high onset potential of the pristine nickel electrocatalyst. On the other hand, as shown in Figure 8, increasing the Mn content more than 40 wt % in the metal nanoparticles decreases both of cell potential and the generated power. Moreover, the pristine Mn nanoparticles showed very low cell potential and power density, however it still better than the pristine nickel nanoparticles-functionalized carbon felt.

Furthermore, it can be seen that the low urea concentration is preferable as the maximum cell potentials and the generated power densities were obtained at a urea solution concentration of 0.5 M. At high concentrations of urea, the anodic current density decreases that may be due to a kinetics of the urea oxidation reaction, this finding matches previous reports studied the urea oxidation reaction [9,13]. This phenomenon explains that the surface coverage of urea molecules on the electrocatalyst becomes high at high concentrations, which in turn decreases the oxidation rate of urea due to the local deprivation of OH^−^ species [13].

## 4. Conclusions

Due to the abnormal decomposition of the nickel and manganese acetates under an inert atmosphere, calcination of carbon felt loaded with these precursors results in the production of a NiMn nanoparticles-functionalized GDL as an ready-made anode to be exploited in direct urea fuel cells. The composition of the deposited metal nanoparticles should be optimized as the performance of the treated carbon felts depends on the composition; the nanoparticles containing 40 wt % Mn reveal the best performance. Interestingly, incorporation of manganese with nickel can modify the proposed nickel-based readymade anode to be workable in the direct urea fuel cells. However, the generated power is not high compared to other types of fuel cells due to the high ohmic resistance and the anode overpotential. Furthermore, the anode overpotential can be diminished by optimization the co-catalyst content; 40 wt % reveals the lowest overpotential which results in maximizing the generated power. Although the generated current density of the proposed NiMn-loaded carbon felt is small compared to several reported materials, the onset potential of the introduced anode material is very acceptable with respect to the corresponding onset potentials of those reported materials that constrain their application in the direct urea fuel cell. In addition, it is recommended to use low urea solution concentration to maximize the generated power density. The functionalization process of the carbon felt is simple and can be carried for different nickel-based electrocatalysts.

## Figures and Tables

**Table 1:** Average resistance of the cells assembled by the prepared GDLs and work with different urea concentrations (kΩ).

Urea Concentration (M)	Mn Content (%)						
	5	10	20	40	60	80	100
0.5	4.59	1.66	3.386	0.896	3.808	3.585	2.488
1.0	3.089	0.685	2.964	0.136	2.739	1.973	2.385
2.0	2.732	2.439	2.135	0.085	2.256	2.244	2.403
